# Heteroresistant Vancomycin Intermediate Coagulase Negative Staphylococcus in the NICU: A Systematic Review

**DOI:** 10.1371/journal.pone.0164136

**Published:** 2016-10-07

**Authors:** Jasmine Chong, Chelsea Caya, Simon Lévesque, Caroline Quach

**Affiliations:** 1 Department of Epidemiology & Biostatistics, McGill University, Montreal, QC, Canada; 2 Laboratoire de santé publique du Québec, Montreal, QC, Canada; 3 Infectious Disease Division, Department of Pediatrics and Department of Medical Microbiology, The Montreal Children’s Hospital of the McGill University Health Centre, Montreal, QC, Canada; Rockefeller University, UNITED STATES

## Abstract

**Context:**

NICUs in the province of Québec have seen an increase in hVICoNS, detected in the clinical laboratory.

**Objective:**

To investigate the clinical relevance of hVICoNS on the course of infection, and to determine the prevalence of hVICoNS sepsis in the NICU.

**Methods:**

We searched MEDLINE, EMBASE, and PubMed from 1 January 1980 to 1 July 2016. Both observational and interventional studies were considered eligible if they provided data on hVICoNS in the NICU population. Two investigators independently reviewed studies for data extraction. Data extracted included: number of CoNS cultures, prevalence of hVICoNS, and clonality of strains.

**Results:**

Of the 613 studies identified, 19 studies were reviewed, and 5 studies included in the final review. No studies addressed the clinical significance of hVICoNS in the NICU. The prevalence of hVICoNS in the NICU varied greatly, ranging from 2.3% to 100%.

**Limitations:**

Publication bias could not be assessed, and risk of bias in some of the included studies due to small sample size and poor methods reporting. The quality of all included studies was low according to GRADE criteria, and the inclusion criteria restricted to either English or French studies.

**Conclusions:**

Our review suggests that heteroresistance to vancomycin is much more common than previously believed. Our search however did not identify any studies that explicitly assessed any clinical implications of hVICoNS infections, thereby highlighting the need for research to assess the true impact of hVICoNS infection and to determine its significance on patient mortality and morbidity in the NICU.

## Introduction

Coagulase-negative staphylococci (CoNS) have emerged as a leading cause of bloodstream infections (BSI) in intensive care units (ICU)[1, 2]. Patients in neonatal intensive care units (NICU) are particularly at risk for healthcare-associated infections (HAI), given their immature immune systems, the acuity of care needed, and the frequency of invasive procedures performed [3, 4]. Though CoNS BSI are not as severe as infections with other pathogens, they lead to increased morbidity, such as a higher relative risk of bronchopulmonary dysplasia in premature infants with CoNS sepsis compared to premature infants without CoNS sepsis [5, 6]. Additionally, associations between CoNS sepsis and neurodevelopmental anomalies, including cerebral palsy have been observed [7, 8]. Infections with CoNS also lead to higher rates of antibiotic use, prolonged hospital stays, and higher healthcare costs [4, 9].

Over 90% of clinical CoNS isolates carry the *mecA* gene, which is associated with beta-lactam antibiotic resistance (methicillin)[10, 11]. Vancomycin is therefore often considered as the first-line antimicrobial therapy. Resistance to vancomycin however, has been identified in clinical isolates of *Staphylococcus aureus* (minimum inhibitory concentration, MIC, >16 mg/L according to CLSI 2016), and reduced susceptibility to vancomycin, a phenomenon dubbed heteroresistance, has been well described in this species (hVISA)[12, 13]. Vancomycin heteroresistance, where there exists a vancomycin-intermediate subpopulation of cells in an otherwise susceptible microbial population, has also been detected in clinical CoNS isolates [12, 14, 15].

Recently, some NICUs have seen an increase in central line associated bloodstream infections (CLABSI) caused by strains of coagulase-negative *Staphylococcus* that developed heterogeneous intermediate resistance to vancomycin (hVICoNS), detected in the clinical laboratory[16]. What remains unclear is the actual clinical relevance of hVICoNS on the course of infection. The primary objective of this systematic review was to determine the clinical relevance of hVICoNS sepsis in patients in the NICU. The secondary objective was to determine the prevalence of hVICoNS sepsis in the NICU population.

## Methods

### Search strategy

We searched MEDLINE, EMBASE, and PubMed from 1 January 1980 to 1 July 2016 to identify research studies on hVICoNS in the NICU. For completeness, we also hand-searched the bibliographies of all initially included studies, though no further studies were found. Details of the search strategy are available in S1 Table.

Two reviewers (JC and CC) independently screened study titles and abstracts for inclusion. In case of disagreement between the two reviewers, a third reviewer was consulted (CQ). Our review was focused on neonatal populations admitted to the NICU with hVICoNS infections; we excluded studies in older children and adults, and studies that only reported hVISA. Studies written in languages other than English or French, and those presented solely as abstracts at scientific conferences were excluded. Studies were also excluded if they focused on colonization and not bacteremia. Both observational and interventional studies were considered eligible if they provided data on hVICoNS in the NICU population (any measure of prevalence). Finally, we accepted any definition of hospital-acquired bacteremia, all types of techniques used for specimen collection, and any approach to hVICoNS screening/detection.

### Data extraction

Following screening, all relevant studies were independently reviewed by two investigators (JC and CC) for data extraction. The following data were extracted: first author, year of publication, country where the study was conducted, study design, study population, population characteristics (sex, age), number of CoNS cultures, number of patients enrolled, type of cultures used for hVICoNS confirmation, vancomycin heteroresistance criteria, and MIC testing method used. In addition, any reported measure of the prevalence of hVICoNS, clonality of hVICoNS strains, and any reported associations with mortality and morbidity were collected. The quality of each included study was assessed independently (JC & CC) using the Grading of Recommendations, Assessment, Development and Evaluation (GRADE) approach.

## Results

### Study selection

**Fig 1:**
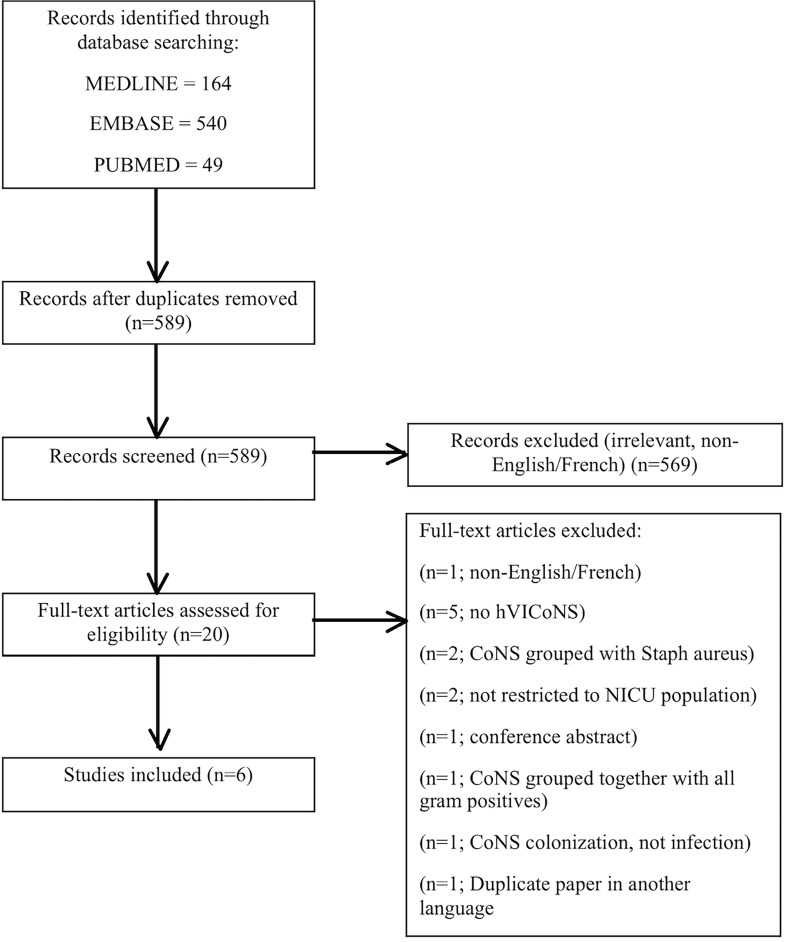
PRISMA flow diagram of identification, screening, eligibility, and inclusion of studies.

Our search strategy identified 753 records, of which 589 were unique (Fig 1). Of these, 569 were excluded because they did not provide data on hVICoNS bacteremia in the NICU. After a review of 20 selected full-text articles and abstracts, six studies met our inclusion criteria and were included in the present systematic review (Table 1)[13, 17–21]. One included study also examined adult/older pediatric ICU patients, but we excluded that information from the data collection [20].

**Table 1:** Characteristics of included studies of hVICoNS in the NICU.

Study	Country	Study Design	Study Population	Data collection, year or year range
Butin et al. 2016	Australia, Belgium, France and United Kingdom	Retrospective laboratory-based prevalence study. MC	86 *S*. *capitis strains* isolated from NICU patients.	2000–2013
D’mello et al. 2007	Australia	Retrospective laboratory based prevalence study. SC	9 *S*. *capitis* strains isolated from NICU infants <1500g	1998–2002
Rasigade et al. 2012	France	Retrospective laboratory-based prevalence study. MC	527 NICU infants >3 days of age	January 2006—April 2009
Van der Zwet et al. 2002	Netherlands	Retrospective observational cohort study. SC	163 NICU infants	1997–2000
Villari et al. 2000	Italy	Prospective surveillance of nosocomial infections in a NICU. SC.	982 NICU infants, 556 males (56.6%), 426 females (43.4%)	January 1996—December 1998
Zubair et al. 2011	Pakistan	Observational cohort study, prospective. SC	388 NICU infants, 252 males (65%), 136 females (35%)	1st December 2009–31st December 2010

SC = Single center, MC = Multi center

### Characteristics of included trials

Characteristics of included trials are summarized in Table 2. Three studies were conducted in Europe (50%), 1 in Australia (16.6%), 1 in Pakistan (16.6%), and 1 in Europe and Australia (16.6%). All studies were observational and designed as either prospective (2 of 6; 33.3%) or retrospective (4 of 6; 66.6%). In all included studies, isolates of CoNS were collected from blood cultures, with one study also collecting samples from tracheal/bronchial aspirate, urine, cerebrospinal fluid, and purulent exudate [17]. D’mello et al. [18] used 4 screening methods to determine heteroresistance, broth microdilution, Etest, VAN 4 screening, and population analysis profile-area under the curve ratio method (PAP-AUC). Following Wootton et al. [22], strains were determined heteroresistant to vancomycin if PAP-AUC ratios were between 1.0–1.3. Rasigade et al. [20] used the BHI screen agar method to determine vancomycin heteroresistance, where strains were considered heteroresistant if ≥1 droplet plated on the BHI agar plate had ≥2 colonies. Van der Zwet et al. [13] used two methods to confirm vancomycin heteroresistance, the first being BHI agar with vancomycin with an aztreonam disk. Strains with enhanced growth around these disks were considered candidates for heteroresistance. Further, representatives were confirmed for heteroresistance using the PAP method. The authors used a definition of subpopulations resistant to 4 mg of vancomycin per liter at frequencies of 2.8x10^-5^, 1.8x10^-4^, 3.0x10^-5^, 6.5x10^-5^, and 3.4x10^-5^ for determining vancomycin heteroresistance. Villari et al. [17] also used the PAP method to investigate heteroresistance. Finally, Zubair et al. [19] determined vancomycin heteroresistance using disk diffusion, following CLSI 2010 (M100-S20) guidelines.

**Table 2:** Published studies containing findings of isolate source, prevalence based on method of screening/detection, and clonality.

Study	Isolate Source	No (%) CoNS isolates	Screening Method	No (%) hVICoNS isolates collected	Clonality
Butin et al. 2016	Blood	86 *S*. *capitis* isolates	Brain heart infusion (BHI) screen agar method to determine heteroresistance (>/1 droplet had >/ 2 colonies)	100% (12/12 representative clinical isolates)	1 widespread *S*. *capitis* clone across 4 countries.
D’mello et al. 2007	Blood	9 *S*. *capitis* isolates	• Broth microdilution • E test • VAN 4 screeing • PAP analysis	• 11.1% (1/9) • 33.3% (3/9) • 100% (9/9) • 100% (9/9)	N/A
Rasigade et al. 2012	Blood	40 *S*. *capitis* isolates	Brain heart infusion (BHI) screen agar method to determine heteroresistance (>/1 droplet had >/ 2 colonies)	100% (40/40) van resistant or heteroresistant (MIC>2 resistant according to EUCAST 2010 using Etest, those MIC ≤ 2 tested for heteroresistance using the BHI agar)	All methicillin-resistant *S*. *capitis* isolates from NICU patients in France belonged to the same pulsotype
Van der Zwet et al. 2002	Blood	217 CoNS isolates	• BHI agar with vancomycin with a 30-ug aztreonam disk • PAPs	• 22.1% (48/217 strains) • 100% (5/5 representative clinical isolates that were positive by screening)	1 *S*. *capitis* strain remained endemic in the NICU since 1998 and was the causative agent for about 1/3 of CoNS bacteremia cases in the unit
Villari et al. 2000	Blood, tracheal/bronchial aspirate, urine, cerebrospinal fluid, purulent exudate	81 *S*. *epidermidis* isolates (50.6% determined to be involved with infection)	• Disk diffusion methods • PAPs	• 0% • 100%	Four predominant clones
Zubair et al. 2011	Blood	388 CoNS isolates	• Kirby-Bauer disc diffusion method	2.3% (9/388 isolates)	N/A

### Quality assessment

Five of six studies obtained a score of ‘low’ for study quality using the GRADE scale, while one received a score of ‘very low’ (S2 Table). Methodological shortcomings in the paper that received a score of ‘very low’ included a very small sample size (n = 9), unclear sampling methods, and possible risk of selection bias [18].

### Prevalence of reduced vancomycin susceptibility

The six studies included in this review did not address our primary objective, the clinical significance of hVICoNS in the NICU, particularly in terms of how the clinical course of hVICoNS bacteremia differs from vancomycin susceptible CoNS bacteremia. From these studies, only data on the prevalence of CoNS with reduced vancomycin susceptibility were found. D’mello et al. [18] assessed nine *S*. *capitis* isolates, all of which demonstrated heterogeneous resistance to vancomycin, detected through PAP analysis. Rasigade et al. [20] analyzed 40 methicillin-resistant *S*. *capitis* isolates collected from various NICUs in France, of which 62.5% were identified as heteroresistant, and 37.5% were determined resistant (following EUCAST 2010 recommendations). Similarly, Bentin et al. [21] evaluated 86 methicillin-resistant *S*. *capitis* isolates collected from NICUs in France, Belgium, Netherlands, and Australia: 100% of the strains were determined heteroresistant or resistant (following EUCAST 2010 recommendations). Van der Zwet et al. [13] screened 217 CoNS isolates and found that 22.1% of the strains were heteroresistant using the BHI agar method. Using the PAP method, Villari et al. [17] found that all 81 *S*. *epidermidis* isolates displayed heterogeneous resistance to vancomycin. Finally, Zubair et al. [19] assessed 388 CoNS isolates, of which 2.3% demonstrated vancomycin heteroresistance using the disk diffusion method. It should be noted that the prevalence of hVICoNS varied across the studies owing in part to different NICU populations, methods of heteroresistance detection, and number/variety of strains collected.

## Discussion

### Clinical impact and prevalence

The primary objective of this systematic review was to assess the literature on hVICoNS and determine its clinical impact in the NICU. Five of the six studies did not explicitly address the clinical impact of hVICoNS bacteremia in the NICU, but did report prevalence data, which was our secondary objective. The remaining study [13] sought to evaluate whether heteroresistance played a role in the therapeutic failure of a single infant. They reported that the infant, from whom a single CoNS isolate was sampled, died from necrotizing enterocolitis during an episode of sepsis caused by heteroresistant *S*. *capitis*. The authors suspected that heteroresistance and treatment with β-lactam antibiotics might have played a role in vancomycin therapy failure and subsequent death, but state that the true clinical significance of heteroresistant CoNS remains to be elucidated.

While not much is known concerning the clinical significance of hVICoNS, heterogeneous vancomycin-intermediate *Staphylococcus aureus* (hVISA) is known to be associated with persistent bacteremia and vancomycin therapeutic failure (not limited to the NICU population) [23]. For instance, Casapao and colleagues found that infections in patients with hVISA were significantly associated with higher vancomycin treatment failure and longer duration of bacteremia compared to patients with sepsis caused by vancomycin susceptible *S*. *aureus* (VSSA)[24]. Moreover, a systematic review and meta-analysis evaluated the clinical significance of hVISA and found a 2.37 times higher glycopeptide treatment failure rate (95% CI, 1.53 to 3.67) in hVISA infections compared to VSSA infections. No significant differences, however in 30-day mortality have been found between hVISA and VSSA infections [23, 24]. These clinically relevant associations found in patients with hVISA sepsis can be used as a starting point for what can be learned about hVICoNS infections, particularly in the NICU.

### Screening methods

Methods of screening/detection of vancomycin heteroresistance were not uniform among the included papers. For instance, the modified Etest (BHI agar) was used in three of the included studies [13, 20, 21]. Further, the population analysis profile-area under the curve ratio method (PAP-AUC), the current gold standard, was used in three studies to determine vancomycin intermediate heteroresistance in CoNS strains [13, 17, 18]. Using this method, all three studies identified vancomycin heteroresistance among all assessed strains [13, 17, 18]. Finally, Zubair and colleagues only used the disk diffusion method to detect decreased vancomycin susceptibility in their study [19]. This method is however no longer considered acceptable to correctly detect heteroresistance [25]. Furthermore, these authors followed the CLSI 2010 (M100-S20) guidelines, which recommended at that time that disk diffusion was not a suitable method to determine vancomycin resistance. The use of the modified Etest and/or PAP method instead may have detected far more hVICoNS among the collected isolates.

Standardization of methods to detect vancomycin intermediate heteroresistance amongst CoNS is necessary to have a clear and correct picture of hVICoNS prevalence that is comparable amongst various clinical settings. While the PAP method is the most reliable and is considered the gold standard, it is much too labour intensive for routine purposes and is not currently feasible in terms of resources and time. Therefore the method used in some included papers, as recommended by Walsh and colleagues [26], of performing modified Etests and then confirming these results with the PAP method, is a suitable alternative to performing PAP analyses for all strains [13, 17, 18]. The sensitivity of the modified Etest as compared to the PAP-AUC is 82 or 96%, and the specificity is 93 or 97%, dependent on the McFarland inoculum used (0.5 or 2.0 respectively)[26]. The modified Etest (BHI agar) is far more reliable than standard laboratory methods with higher sensitivity and specificity when detecting heteroresistance and has been proven to be a valid substitute to PAP [26]. Utilizing such a method to determine vancomycin intermediate heteroresistance will result in a much more accurate and standardized overview of hVICoNS prevalence in the clinical setting.

### Clonality

Clonality was evaluated in four of the six included studies. Van der Zwet and colleagues concluded that the 48 hVICoNS (of 217) strains were *S*. *capitis* and that there was a clonal spread of a single *S*. *capitis* strain that remained endemic in the NICU from 1998 to 2000 [13]. Rasigade and colleagues determined that all of the methicillin-resistant *S*. *capitis* isolates that were collected from various NICUs in France belonged to the same pulsotype (NRSC-A). These findings indicated a clonal spread of methicillin-resistant *S*. *capitis* with reduced vancomycin susceptibility in various French NICUs [20]. Further, Butin et al. uncovered the widespread clonal spread of the same *S*. *capitis* clone across European and Australian NICUs [21]. Villari and colleagues identified four predominant clones among a total of 28, which they found to be more antibiotic resistant than the other clones. Moreover, they determined that strains from the four predominant clonal groups included the strains where growth was inhibited by the highest concentrations of glycopeptides [17].

These findings of successful heteroresistant clones in the NICU suggest that reduced glycopeptide susceptibility may play a role in their prolonged persistence in a clinical setting. Among *S*. *capitis* strains in particular, it has been suggested that vancomycin heteroresistance is an intrinsic property of this particular species, and that increased pressure from vancomycin therapy in the NICU resulted in its selection and success [18, 20]. Evidently, the widespread geographical spread and propagation of clinical hVICoNS strains will be of concern if detrimental clinical consequences are uncovered.

### Strengths and weaknesses

The main strength of this systematic review is that it used a comprehensive search strategy that reviews the current state of hVICoNS in the NICU. Additionally, we conducted the review according to a pre-specified protocol (S3 Table).

Limitations were also present in this review, the first being an insufficient number of studies to adequately interpret a funnel plot to assess the presence of publication bias. Other limitations include risk of bias in some of the included studies due to small sample size and poor methods reporting (mainly the process of selecting participants). The quality of all included studies was low according to the GRADE scale, as they were all observational, and the inclusion criteria restricted to either English or French studies, potentially creating bias. Moreover, the primary aim of five of the six included studies was not to assess the clinical impact of hVICoNS in the NICU in terms of associations with morbidity and mortality. As addressed above, there was methodological heterogeneity, affecting the generalizability and ability to pool hVICoNS prevalence.

## Conclusion

The prevalence of hVICoNS in the NICU varied greatly in the literature, ranging from 2.3% [19] to 100% [17, 18, 20, 21]. This heterogeneity is due in part to differences in the number and species of CoNS strains investigated, clonality of strains tested, as well as the various methods of hVICoNS detection used. Further, a standardized method to detect heteroresistance is required to accurately address the role of hVICoNS in the clinical setting. The data presented in this review gives an idea of the clinical prevalence but does not help elucidate the clinical impact of hVICoNS in the NICU. Our review suggests that heteroresistance to vancomycin is much more common than previously believed. Our search, however, did not identify any studies that explicitly assessed any clinical implications of hVICoNS infections, thereby highlighting the need for research into this topic.

More detailed evaluations are needed to determine the true influence of hVICoNS on the clinical course of infection. Most of the included studies did not collect detailed clinical information from patients, and population characteristics were not separated into hVICoNS and vancomycin susceptible CoNS (VSCoNS). This must be remedied in future studies in order to better understand the clinical relevance of hVICoNS in the NICU. Ideally, this research would be completed prospectively and hVICoNS detection would follow the recommendations of Walsh et al.[26]. These future studies could determine whether hVICoNS infection are associated with prolonged septicemia or higher rates of vancomycin therapeutic failure, as are hVISA, or if mortality rates differ between hVICoNS and VSCoNS.

Whether infection caused by vancomycin heteroresistant CoNS significantly differs from those of VSCoNS may have implications as to how CoNS are currently treated in the NICU. That is, if hVICoNS are found to be associated with glycopeptide treatment failure, then other treatments should be given in replacement of vancomycin after reduced glycopeptide susceptibility is determined in the causative bacteremic agent. Furthermore, the presence of a vancomycin intermediate resistant subpopulation in the cells may be of concern as it could lead to future vancomycin resistance. Therefore, adherence to hospital infection control practices and surveillance of CoNS vancomycin MICs are necessary to avoid vancomycin resistance.

The findings from this systematic review underscore the need for more studies to better comprehend the clinical relevance of hVICoNS infection in the NICU. Apart from a single study where vancomycin heteroresistance, according to the authors, may have played a role in vancomycin therapeutic failure in a single infant, there are no studies that even aim to investigate the role of heteroresistance in CoNS bacteremia. Further research and a standardized means of testing for heteroresistance is necessary to assess the true impact of hVICoNS infection and to determine its significance on patient mortality and morbidity in the NICU.

## Supporting Information

S1 Table Search strategy. (DOCX)

S2 Table Quality of evidence using GRADE. (DOCX)

S3 Table PRISMA checklist. (PDF)
