# Supplementary material for: Heteroresistant Vancomycin Intermediate Coagulase Negative Staphylococcus in the NICU: A Systematic Review
Source: PLoS One. 2016 Oct 7;11(10):e0164136. doi: 10.1371/journal.pone.0164136 (PMC5055326; doi:10.1371/journal.pone.0164136)
Supplement: S1 Table — (DOCX) [file pone.0164136.s001.docx]

Appendix 1. Search strategy

| Search: hVICoNS in the NICU | Search: hVICoNS in the NICU | Search: hVICoNS in the NICU |
| --- | --- | --- |
| Database: Pubmed | Database: EMBASE + Classic EMBASE | Database: Medline |
| ((((((neonatal intensive care units[MeSH Terms]) OR ("Intensive Care, Neonatal"[Mesh] OR "Intensive Care Units, Neonatal"[Mesh])) OR nicu[Text Word])) AND ((((((Staphylococcus cohnii[title/abstract]) OR Staphylococcus caprae[title/abstract]) OR Staphylococcus capitis[title/abstract]) OR Staphylococcus warneri[title/abstract])) OR (("Staphylococcus lugdunensis"[Mesh] OR "Staphylococcus hominis"[Mesh] OR "Staphylococcus haemolyticus"[Mesh] OR "Staphylococcus epidermidis"[Mesh] OR "Staphylococcus saprophyticus"[Mesh] OR "Staphylococcus"[Mesh])))) AND (((((resistan*) OR susceptibility) OR heteroresist*) OR reduced susceptibility) OR intermediate susceptibility)) AND ((((intermediate vancomycin susceptibility) OR Vancomycin Resistance[MeSH Terms]) OR Vancomycin[MeSH Terms]) OR vancomycin[Text Word]) | 1. neonatal intensive care unit.mp.  2. newborn intensive care/  3. newborn sepsis/  4. nicu.mp.  5. newborn/  6. 1 or 2 or 3 or 4 or 5  7. Staphylococcus warneri/ or Staphylococcus capitis/ or staphylococcus.mp. or Staphylococcus cohnii/ or Staphylococcus epidermidis/ or Staphylococcus haemolyticus/ or coagulase negative Staphylococcus/ or Staphylococcus lugdunensis/ or Staphylococcus hominis/ or Staphylococcus caprae/ or Staphylococcus saprophyticus/  8. (epidermidis or warneri or haemolyticus or capitis or caprae or cohnii or hominis or lugdunensis or saprophyticus).mp.  9. Staphylococcus infection/  10. staphylococcal bacteremia/  11. 7 or 8 or 9 or 10  12. antibiotic resistance/  13. (heteroresist* or susceptibility or resistance).mp.  14. antibiotic sensitivity/  15. 12 or 13 or 14  16. vancomycin.mp. or vancomycin/  17. 6 and 11 and 15 and 16 | 1. Intensive Care Units, Neonatal/  2. Intensive Care, Neonatal/  3. nicu.mp.  4. (intensive care adj10 (neonat* or newborn* or new born*)).mp.  5. newborn.mp. or Infant, Newborn/  6. 1 or 2 or 3 or 4 or 5  7. Staphylococcal Infections/  8. Staphylococcus/  9. coagulase negative staph*.mp.  10. staphylococci.mp.  11. staphylococcus epidermidis.mp.  12. (epidermidis or staphylococcus warneri or warneri or staphylococcus haemolyticus or haemolyticus or staphylococcus capitis or capitis or staphylococcus caprae or caprae or staphylococcus cohnii or cohnii or staphylococcus hominis or hominis or staphylococcus lugdunesis or lugdunesis or staphylococcus saprophyticus or saprophyticus).mp.  13. Staphylococcus warneri/ or Staphylococcus capitis/ or Staphylococcus cohnii/ or Staphylococcus epidermidis/ or Staphylococcus haemolyticus/ or Staphylococcus lugdunensis/ or Staphylococcus caprae/ or Staphylococcus hominis/ or Staphylococcus saprophyticus/ or Staphylococcus/  14. 7 or 8 or 9 or 10 or 11 or 12 or 13  15. (heteroresist* or susceptibility or resistance).mp.  16. Vancomycin/  17. Vancomycin Resistance/  18. vancomycin.mp.  19. 16 or 17 or 18  20. 6 and 14 and 15 and 19 |
