# Supplementary material for: Heteroresistant Vancomycin Intermediate Coagulase Negative Staphylococcus in the NICU: A Systematic Review
Source: PLoS One. 2016 Oct 7;11(10):e0164136. doi: 10.1371/journal.pone.0164136 (PMC5055326; doi:10.1371/journal.pone.0164136)
Supplement: S2 Table — (DOCX) [file pone.0164136.s002.docx]

Table: Quality of evidence using GRADE

| Study | Quality of evidence (GRADE) |
| --- | --- |
| Butin et al. 2016 | Low |
| D’mello et al. 2007 | Very Low |
| Rasigade et al. 2012 | Low |
| Van der Zwet et al. 2002 | Low |
| Villari et al. 2000 | Low |
| Zubair et al. 2011 | Low |
